# Robotic Kidney Transplantation from a Brain-Dead Deceased Donor in a Patient with Autosomal Dominant Polycystic Kidney Disease: First Case Report

**DOI:** 10.1089/cren.2018.0050

**Published:** 2018-08-01

**Authors:** Graziano Vignolini, Francesco Sessa, Isabella Greco, Alessandro Pili, Saverio Giancane, Arcangelo Sebastianelli, Giampaolo Siena, Mauro Gacci, Vincenzo Li Marzi, Riccardo Campi, Sergio Serni

**Affiliations:** Department of Urologic Robotic Surgery and Renal Transplantation, University of Florence, Careggi Hospital, Florence, Italy.

**Keywords:** autosomal dominant polycystic kidney disease, case report, kidney transplantation, robotics, brain-dead deceased donor

## Abstract

***Background:*** Autosomal dominant polycystic kidney disease (ADPKD) is a common cause of end-stage renal disease (ESRD) and may pose significant technical challenges for kidney transplantation. Recently, robot-assisted kidney transplantation (RAKT) has been shown to achieve excellent patient and graft outcomes while reducing surgical morbidity. However, the vast majority of RAKT performed so far were from living donors and no studies reported the outcomes of RAKT in patients with ADPKD.

***Case Presentation:*** Herein, we describe the first successful case of RAKT from a brain-dead deceased donor in a 37-year-old patient with ESRD due to ADPKD.

***Conclusion:*** Our case highlights that RAKT can be safely performed by experienced robotic surgeons even in selected complex recipients such as patients with ADPKD and using grafts from deceased donors.

## Introduction

Kidney transplantation (KT) represents the most effective treatment in patients with end-stage renal disease (ESRD).

Autosomal dominant polycystic kidney disease (ADPKD) is a common cause of ESRD and may pose significant technical challenges for KT.^1^

Despite there is still controversy regarding indications and timing of native kidney nephrectomy (NKN), recent evidence suggests that in asymptomatic ADPKD patients, especially with residual diuresis, NKN before or at the time of KT should not be performed in the absence of space conflicts, as it may increase the risk of adverse postoperative outcomes.^1^

In recent years, robot-assisted kidney transplantation (RAKT) has been shown to accurately reproduce the principles of open KT achieving excellent outcomes while reducing surgical morbidity.^2^

However, the vast majority of RAKTs were performed from living donors and no studies reported the outcomes of RAKT in patients with ADPKD.

Herein, we describe the first case of RAKT with regional hypothermia from a brain-dead deceased donor in a 37-year-old patient with ESRD due to ADPKD.

## Case Report

### Clinical case

A 37-year-old Caucasian male patient was referred to our Department from the National Organ Procurement and Transplant Network waiting list for KT.

The recipient was an unmarried office worker with hypertension under pharmacological treatment, no previous abdominal surgery and ESRD due to ADPKD currently not requiring hemodialysis (preemptive). Pretransplant estimated glomerular filtration rate (eGFR) was 13.7 mL/minute per 1.73 m^2^. Body mass index was 21.9 kg/m^2^. At physical examination, an asymptomatic bilateral flank mass was palpable.

**FIG. 1.**
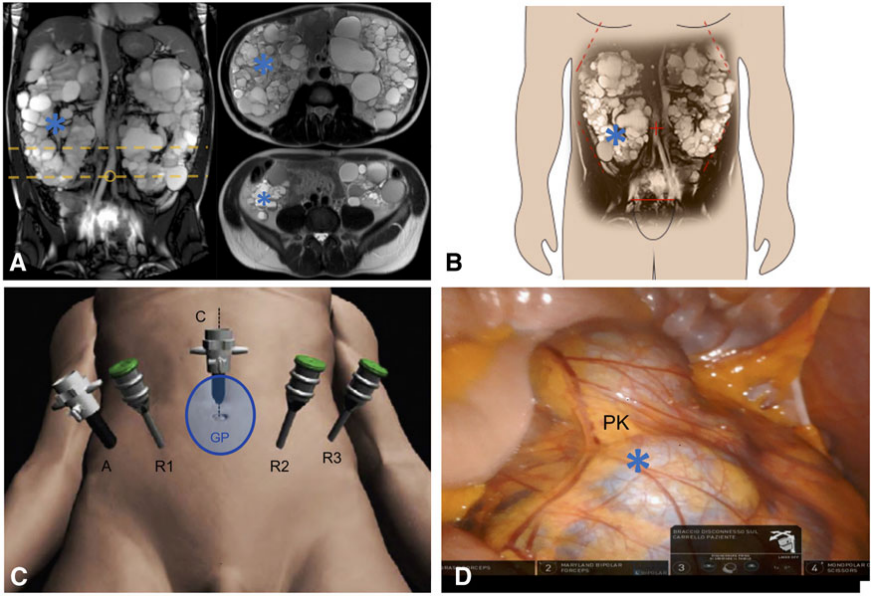
Overview of robotic kidney transplantation in our case. **(A,** **B)**. MRI images showing the voluminous polycystic native kidneys. No space limitations at the level of transplantation site at the right iliac fossa were noted. The *yellow dotted lines* in **A** indicate the level of the transversal sections of the MRI images showed on the *right* side of **A**. **(C)** Trocar placement. A 4- to 5-cm midline periumbilical incision was made for the GelPOINT access device. A pneumoperitoneum of 12 mm Hg was established, and three 8 mm robotic ports and one 12 mm additional assistant port were inserted under vision in a modified RARP configuration. All trocars were positioned ∼3 cm downward on the same lines to obtain an increased working space far from the enlarged PKs. The patient was then turned to a 30° Trendelenburg position and the da Vinci Xi Robot^®^ was docked on the lateral patient side. **(D)** Intraoperative snapshot showing the distal portion of the enlarged right PK (*): no space constraints were present at the level of the right iliac fossa. PK, polycystic kidney; RARP, robot-assisted radical prostatectomy.

Abdominal MRI scan confirmed the presence of multiple cysts of different diameter within significantly enlarged native kidneys, whose sagittal diameter was >25 cm bilaterally (Fig. 1A). No space constraints were anticipated before RAKT at the putative transplantation site (right iliac fossa) (Fig. 1B, C). As such, no indication was placed for NKN.

The brain-dead donor was a 50-year-old Asiatic male, without significant comorbidities, deceased in a motor vehicle accident. At CT scan, a single renal artery and vein were noticed. Surgical technique of abdominal organ procurement followed established surgical principles.^3^ For KT, the right kidney was assigned to our Institution.

### RAKT: surgical technique

After multidisciplinary board discussion and obtaining the informed consent, the patient was scheduled for RAKT.

A step-by-step overview of surgical technique of RAKT used in our case is described in Supplementary Data, as well as in Supplementary Video S1 accompanying the article.

RAKT was performed using the da Vinci Xi Robot^®^ (Intuitive Surgical, Sunnyvale, CA) in a four-arm configuration using a 0° lens with the patient in a 30° Trendelenburg position. Surgical technique followed the principles of the Vattikuti–Medanta technique^2^ with specific technical modifications to tailor the surgical strategy to the specific patient's anatomy (Supplementary Data).

At the beginning of the procedure, the abdominal cavity was inspected to ensure the adequacy of the working space for subsequent transplantation at the right iliac fossa (Fig. 1D).

After exposure of iliac vessels and creation of an extraperitoneal pouch, the bladder was detached and prepared for subsequent ureteroneocystostomy. At this point, the graft within the gauze jacket was introduced into the abdominal cavity through the GelPOINT device. Regional hypothermia was achieved by cooling the graft with 200–250 cc of ice slush introduced through the GelPOINT.^2^

**FIG. 2.**
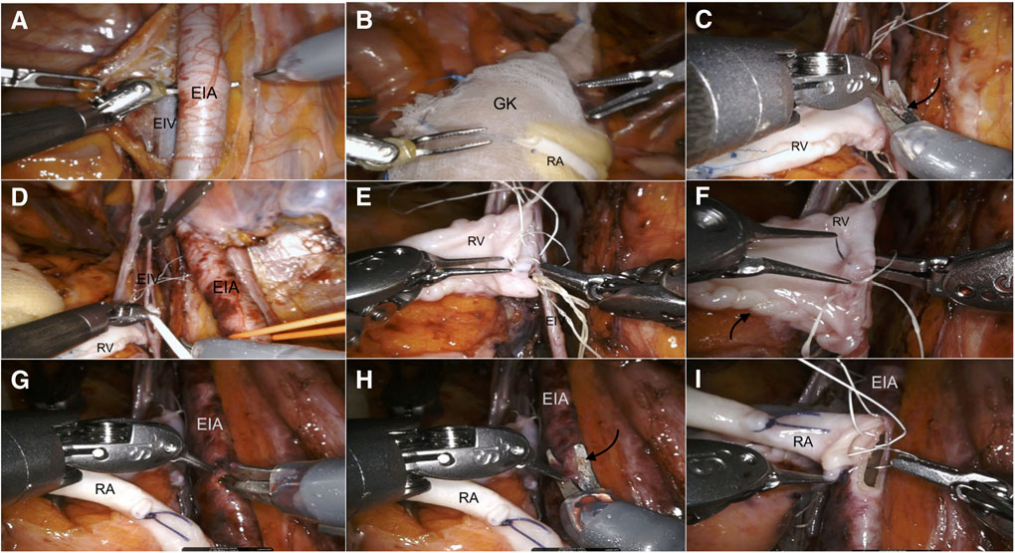
Intraoperative snapshots showing the step-by-step technique for vascular anastomoses during RAKT in our case. RAKT was performed by a dedicated robotic transplant team. Bench preparation of the graft included defatting, perfusion with Celsior^®^ solution, vessel preparation, and preplacement of a 6F, 14 cm DJ. The graft was then inserted into a gauze jacket filled with ice. **(A)** Skeletonization of external iliac vessels. The dissection was more extended in our case of RAKT to ensure avoidance of atherosclerotic plaques at the site of arterial anastomosis. **(B)** Insertion of the graft through the GelPOINT device without need of redocking. **(C)** Venous anastomosis. A venotomy (*arrow*) was performed using cold scissors. **(D)** The lumen of EIV was flushed with heparinized saline before anastomosis. **(E, F)** A running suture from the 12- to 6-o'clock position was performed to close the posterior plate using the needle driver on the surgeon's dominant hand and the Black diamond microforceps on the nondominant hand **(E)**; then, a knot was tied and the anterior plate was completed with the same suture from the 6- to 12-o'clock position **(F)**. **(G, H)** Linear arteriotomy and its conversion to circular arteriotomy using cold scissors. **(I)** Arterial anastomosis. Two half-running sutures starting at the 12 o'clock position and running toward the 6 o'clock position were performed with two threads to close the posterior and then anterior plates of the anastomosis. DJ, Double-J stent; EIA, external iliac artery; EIV, external iliac vein; GK, graft kidney; RA, renal artery; RAKT, robot-assisted kidney transplantation; RV, renal vein.

After a venotomy was made using a curved cold scissors, the graft renal vein was anastomosed in an end-to-side continuous fashion to the external iliac vein using a 6/0 Gore-Tex suture on a CV-6 TTc-9 needle (Fig. 2). After completion of the venous anastomosis, additional ice slush was introduced to ensure graft cooling.

Subsequently, after clamping of the external iliac artery, a linear arteriotomy was made using robotic curved cold scissors. Then, the arteriotomy was modeled as a circular arteriotomy and the lumen was flushed with heparinized saline. Then, the renal artery was anastomosed in an end-to-side continuous fashion to the external iliac artery using a 6/0 Gore-Tex suture on a CV-6 TTc-9 needle (Fig. 2). After testing the integrity of the anastomosis, the kidney was revascularized. The graft was inspected for color and turgor and pneumoperitoneum was reduced to 8 mm Hg to check for any bleeding.

**FIG. 3.**
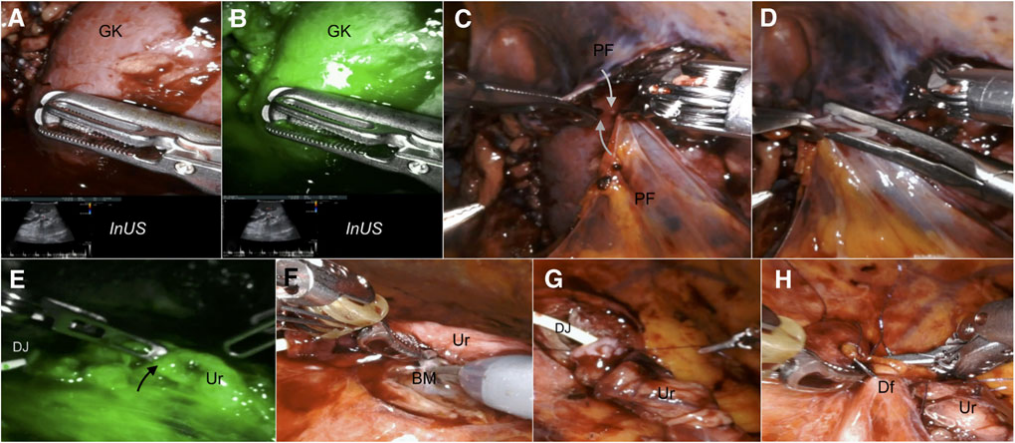
Intraoperative snapshots showing graft reperfusion and the key steps of ureterovesical anastomosis in our case. **(A, B, E)**. After venous injection of 0.3 mg/kg of indocyanine green, Firefly^®^ fluorescence imaging technology and InUS were used to check graft reperfusion and integrity of ureteral vascularization (*black arrow*). **(C, D)**. Closure of the extraperitoneal pouch reapproximating the two previously prepared PF by using hem-o-Lok clips (*white arrows*). **(F–H)**. Key steps of ureteroneocystostomy using a modified Lich-Gregoir technique. After spatulation, the Ur is anastomosed to the bladder mucosa in a continuous fashion over the preplaced DJ; then, the detrusor muscle was closed with a running suture creating an antirefluxing mechanism. BM, bladder mucosa; Df, detrusor flap; InUS, intraoperative duplex ultrasound; PF, peritoneal flaps; Ur, ureter.

Firefly^®^ fluorescence imaging technology was used to check kidney and ureteral vascularization in conjunction with intraoperative duplex ultrasound using the da Vinci TilePro™ feature (Fig. 3).

The kidney was then allocated into the extraperitoneal pouch. Finally, the ureterovesical anastomosis was performed using a modified Lich-Gregoir technique using two semicontinuous sutures with 4-0 Monocryl sutures (Ethicon, Inc, Cincinnati, OH) over the preplaced 6F, 14 cm Double-J stent. The detrusor muscle was closed with a continuous suture creating an antirefluxing mechanism (Fig. 3).

Intraoperative and perioperative outcomes are shown in Table 1.

**Table 1.** Intraoperative and Postoperative Outcomes After Robot-Assisted Kidney Transplantation in Our Case

Intraoperative outcomes	
Overall operative time (minutes)	200
Console time (minutes)	150
Venous anastomosis time (minutes)	26
Arterial anastomosis time (minutes)	15
Ureterovesical anastomosis time (minutes)	9
Cold ischemia time (hours)	15
Rewarming time (minutes) (time from introduction of the graft into the abdominal cavity under regional hypothermia to graft revascularization)	57
Estimated blood loss (mL)	100
Intraoperative complications	0
Postoperative outcomes	
eGFR at postoperative day 1 (mL/minute per 1.73 m^2^)	16.7
eGFR at postoperative day 3 (mL/minute per 1.73 m^2^)	35.1
eGFR at postoperative day 7 (at discharge) (mL/minute per 1.73 m^2^)	53.1
Length of hospitalization (days)	7
Double-J stent removal (POD)	28
Follow-up (months)	6
Postoperative complications	0
eGFR at follow-up (6 months) (mL/minute -per 1.73 m^2^)	76.8

eGFR = estimated glomerular filtration rate (CKD-EPI 2009 equation); POD = postoperative day.

Duplex ultrasound of the graft performed on postoperative day 1 showed normal graft perfusion.

At 6-month follow-up, the patient was free of symptoms, with regular voiding function, absence of lymphocele at abdominal ultrasound and optimal renal function (eGFR 76.8 mL/minute per 1.73 m^2^).

## Discussion

To the best of our knowledge, this is the first case of RAKT from a brain-dead deceased donor in a recipient with ADPKD.

Thanks to the advantages of the robotic platform and the opportunity to use fluorescence imaging to check graft and ureteral reperfusion, RAKT allows to improve precision and technical finesse of vascular anastomoses replicating the principles of open, while reducing surgical morbidity, which is key for fragile and immunocompromised patients with ESRD.^2^

In our case, RAKT was successfully performed using a standardized surgical technique previously employed for living donors robotic transplantations^2^ with specific technical modifications aiming to tailor the surgical strategy to the specific patient's anatomy.

First, dissection of iliac vessels was slightly more extended as compared with RAKT from living donors; second, a higher Trendelenburg tilt (30° *vs* 15–20°) and pneumoperitoneum pressure (12 *vs* 10 mm Hg), as well as a more caudal trocar placement, were needed to increase the working space and the distance from the voluminous polycystic native kidneys.

A recent study showed that surgeons with extensive robotic experience had only *minimal* learning curve for RAKT or *no* learning curve *at all* depending on their previous experience in open KT.^4^ As such, adoption of RAKT is likely to increase in the future especially at referral Centers with experience in both robotic urologic surgery and open KT.

Overall, our case highlights that RAKT can be safely performed by experienced robotic surgeons even in selected complex recipients such as patients with ADPKD and using grafts from deceased donors.

Larger studies with longer follow-up and appropriate study design are needed to confirm feasibility and safety of RAKT in these complex clinical scenarios.

## Supplementary Material

Supplemental data

Supplemental data

## Abbreviations Used

ADPKD: autosomal dominant polycystic kidney disease
CT: computed tomography
Df: detrusor flap
eGFR: estimated glomerular filtration rate
EIA: external iliac artery
EIV: external iliac vein
ESRD: end-stage renal disease
GK: graft kidney
InUS: intraoperative duplex ultrasound
KT: kidney transplantation
MRI: magnetic resonance imaging
NKN: native kidney nephrectomy
PF: peritoneal flaps
PK: polycystic kidney
POD: postoperative day
RA: renal artery
RAKT: robot-assisted kidney transplantation
RARP: robot-assisted radical prostatectomy
RV: renal vein
Ur: ureter
