# Supplementary material for: Robotic Kidney Transplantation from a Brain-Dead Deceased Donor in a Patient with Autosomal Dominant Polycystic Kidney Disease: First Case Report
Source: J Endourol Case Rep. 2018 Aug 1;4(1):124–8. doi: 10.1089/cren.2018.0050 (PMC6083208; doi:10.1089/cren.2018.0050)
Supplement: Supplemental data [file Supp_Data.pdf]

Supplementary Data

SUPPLEMENTARY VIDEO S1. Step-by-step technique for robotic kidney transplantation in our case.

Supplementary Details on Surgical Technique of Robotic Kidney Transplantation in Our Case

After induction of general anesthesia, a 4- to 5-cm midline periumbilical incision was made for the GelPOINT access device. A 12-mm camera port was placed into the GelSeal cap of the GelPOINT device (GP) as previously described.[S1] A pneumoperitoneum of 12 mm Hg was established, and three 8-mm robotic ports and one 12 mm additional assistant port were inserted under vision in a modified robot-assisted radical prostatectomy configuration.[S1] All trocars were positioned ~3 cm downward on the same lines to obtain an increased working space far from the enlarged polycystic kidneys. The patient was then turned to a 30° Trendelenburg position and the da Vinci Xi Robot® was docked on the lateral patient side.

Robot-assisted kidney transplantation (RAKT) was performed by a dedicated robotic transplant team, including one senior transplant surgeon with extensive experience in both open kidney transplantation and robotic urologic surgery [G.V.] and two bed-side assistants experienced in laparoscopic surgery [A.S. and F.S.].

Open bench preparation of the graft included defatting, perfusion with Celsior® solution, vessel preparation (including reconstruction of the right renal vein with a caval patch) and preplacement of a 6F, 14 cm Double-J stent into the urinary collecting system. The graft was then inserted into a gauze jacket filled with ice with stay sutures on renal artery to orientate the graft during RAKT.

The key steps of the procedure are highlighted below:

Step 1: Skeletonization of iliac vessels. The dissection of iliac vessels was more extended in this case of RAKT from a deceased donor compared with previous living donor cases to ensure avoidance of atherosclerotic plaques at the site of arterial anastomosis.

Step 2: Insertion of the graft through the GelPOINT device. After the GelSeal cap was repositioned and the camera redocked, the graft was oriented to achieve optimal exposure of the vessels for vascular anastomoses.

Step 3: Venous anastomosis. Robotic bulldog clamps were used to clamp the external iliac vein. A venotomy (arrow) was performed using cold scissors. The lumen of external iliac vein was flushed with heparinized saline through a 5F ureteric catheter introduced through the assistant port before venous anastomosis. A running suture from the 12- to 6-o'clock position was performed to close the posterior plate of the venous anastomosis using the needle driver on the surgeon's dominant hand and the Black diamond microforceps on the nondominant hand; then, a knot was tied at the 6 o'clock position and the anterior plate of venous anastomosis was completed with a running suture using the same suture from the 6- to 12-o'clock position.

Step 4: Arterial anastomosis. A linear arteriotomy was made using cold robotic scissors. Then, it was converted into a circular arteriotomy using robotic scissors. Two half-running sutures (both starting at 12 o'clock position and running toward 6 o'clock position) were performed to close first the posterior and then the anterior plates of the arterial anastomosis.

Step 5: Graft reperfusion. After venous injection of 0.3 mg/kg of indocyanine green, fluorescence imaging technology and intraoperative duplex ultrasound were used to check graft reperfusion and integrity of ureteral vascularization.

Step 6: Ureterovesical anastomosis in our case. The extraperitoneal pouch was closed reapproximating the two previously prepared peritoneal flaps by using hem-o-Lok clips. Ureteroneocystostomy was performed using a modified Lich-Gregoir technique. After spatulation of the ureter for ~1 cm, the ureter is anastomosed to the bladder mucosa in a continuous fashion over the preplaced Double-J stent; then, the detrusor muscle was closed with a running suture creating an antirefluxing mechanism.

Supplementary Reference

S1. Menon M, Sood A, Bhandari M, et al. Robotic kidney transplantation with regional hypothermia: A step-by-step description of the Vattikuti Urology Institute–Medanta Technique (IDEAL Phase 2a). Eur Urol 2014;65:991–1000.
